# Spatiotemporal heterogeneity and its determinants of COVID-19 transmission in typical labor export provinces of China

**DOI:** 10.1186/s12879-021-05926-x

**Published:** 2021-03-05

**Authors:** Li Wang, Chengdong Xu, Jinfeng Wang, Jiajun Qiao, Mingtao Yan, Qiankun Zhu

**Affiliations:** 1 College of Environment and Planning, Henan University, KaiFeng, 475001 China; 2 Key Laboratory of Geospatial Technology for the Middle and Lower Yellow River Regions (Henan University), Ministry of Education, KaiFeng, 475001 China; 3 State Key Laboratory of Resources and Environmental Information System, Institute of Geographic Science and Natural Resource Research, Chinese Academy of Sciences, Beijing, 100101 China

**Keywords:** COVID-19, Labor export region, Spatiotemporal pattern, Socioeconomic risk factors

## Abstract

**Background:**

Previous studies have indicated that the risk of infectious disease spread is greatest in locations where a population has massive and convenient access to the epicenter of an outbreak. However, the spatiotemporal variations and risk determinants of COVID-19 in typical labor export regions of China remain unclear. Understanding the geographical distribution of the disease and the socio-economic factors affecting its transmission is critical for disease prevention and control.

**Methods:**

A total of 2152 COVID-19 cases were reported from January 21 to February 24, 2020 across the 34 cities in Henan and Anhui. A Bayesian spatiotemporal hierarchy model was used to detect the spatiotemporal variations of the risk posed by COVID-19, and the GeoDetector *q* statistic was used to evaluate the determinant power of the potential influence factors.

**Results:**

The risk posed by COVID-19 showed geographical spatiotemporal heterogeneity. Temporally, there was an outbreak period and control period. Spatially, there were high-risk regions and low-risk regions. The high-risk regions were mainly in the southwest areas adjacent to Hubei and cities that served as economic and traffic hubs, while the low-risk regions were mainly in western Henan and eastern Anhui, far away from the epicenter. The accessibility, local economic conditions, and medical infrastructure of Wuhan in Hubei province all played an important role in the spatiotemporal heterogeneity of COVID-19 transmission. The results indicated that the *q* statistics of the per capita GDP and the proportion of primary industry GDP were 0.47 and 0.47, respectively. The *q* statistic of the population flow from Wuhan was 0.33. In particular, the results showed that the *q* statistics for the interaction effects between population density and urbanization, population flow from Wuhan, per capita GDP, and the number of doctors were all greater than 0.8.

**Conclusions:**

COVID-19 showed significant spatiotemporal heterogeneity in the labor export regions of China. The high-risk regions were mainly located in areas adjacent to the epicenter as well as in big cities that served as traffic hubs. Population access to the epicenter, as well as local economic and medical conditions, played an important role in the interactive effects of the disease transmission.

**Supplementary Information:**

The online version contains supplementary material available at 10.1186/s12879-021-05926-x.

## Background

A novel coronavirus infection (COVID-19) caused by SARS-CoV-2 was first identified in Wuhan of the Hubei province in China in December 2019 [1]. As of January 26, 2021, COVID-19 has resulted in a total of 2,124,193 people dead in the world [2]. The COVID-19 pandemic has caused a massive health crisis worldwide and has had a huge impact on the global social economy, transportation, politics, diplomacy, and more [3].

Although China launched emergency control measures early in the outbreak, including a travel quarantine for Wuhan, movement restrictions, extended holidays, canceled crowd gatherings, calls for home isolation, and more [4, 5], the COVID-19 pandemic had already affected hundreds of thousands of people leaving Wuhan during the first two weeks of the Spring Festival transport season, many of whom potentially carried and spread the novel coronavirus to their destination regions, leading to a countrywide health challenge [6–8].

Accurately identifying epidemic trends in advance can reveal much about the geographic risks and socio-economic factors impacting the transmission mechanism of a new coronavirus, as well as how to respond to it. Recently, many studies have focused on predicting the epidemic trend of COVID-19 [8–12]. In the early stages, Joseph used the SEIR model to forecast the national and global spread risks of COVID-19 based on human mobility data in 300 prefecture-level cities of China [13]. In order to make the epidemic forecast more closely fit the actual situation, some scholars applied a well-mixed SEIR model and exposed-identified-recovered (EIR) model to assess the epidemic spreading processes from the free propagation phase to the extremely controlled phase [12, 14]. Other scholars used a stochastic transmission model, Bayesian model, or another mathematical model to assess the effects of intervention policies on the spread of imported cases [11, 15, 16]. In addition, the increased spatial stratified heterogeneity of the disease spread has been studied to clarify the characteristics of the epidemic [11, 17]. Though attempts have been made to improve the accuracy and validity of these estimates, the currently available estimates regarding the domestic and international transmission of COVID-19 are rather inconsistent, because of the spatiotemporal heterogeneity of the disease spread and a limited understanding of its transmission mechanisms.

Since Henan and Anhui provinces have the largest migrant outflows in China and are adjacent to Hubei, which was the epidemic outbreak center during the Spring Festival transport season, the return of the hometown population is making these regions the top-ranked hot flow area. The aim of the study was to analyze spatiotemporal heterogeneity and reveal its determinants of COVID-19 transmission in a typical labor export region. A Bayesian spatiotemporal hierarchy model was used to reveal the spatiotemporal heterogeneity of the disease spread. A GeoDetector *q* statistic method was used to detect the risk factors of the disease transmission in the context of its spatial stratified heterogeneity, which can be used to understand the spatiotemporal variations of the disease transmission and is critical for improving future prevention and control measures.

## Materials

### Study region

The Henan and Anhui provinces were selected as the study region. They border the Hubei province where COVID-19 was first identified and were the center of the epidemic in China. These two provinces also have large agricultural populations, from which seasonal or long-term surplus populations outflow to neighboring provinces.

Henan province is located in central China, bound to the south by Hubei, with a total area of 167,000 km^2^. It is a populous province with a permanent population of 96.4 million and a floating population of 12.56 million. Anhui province is bound to the west by the provinces of Henan and Hubei, with a total area of 140,100 km^2^. It has a permanent population of 63.659 million and a floating population of 8.04 million.

### Data

**Fig. 1:**
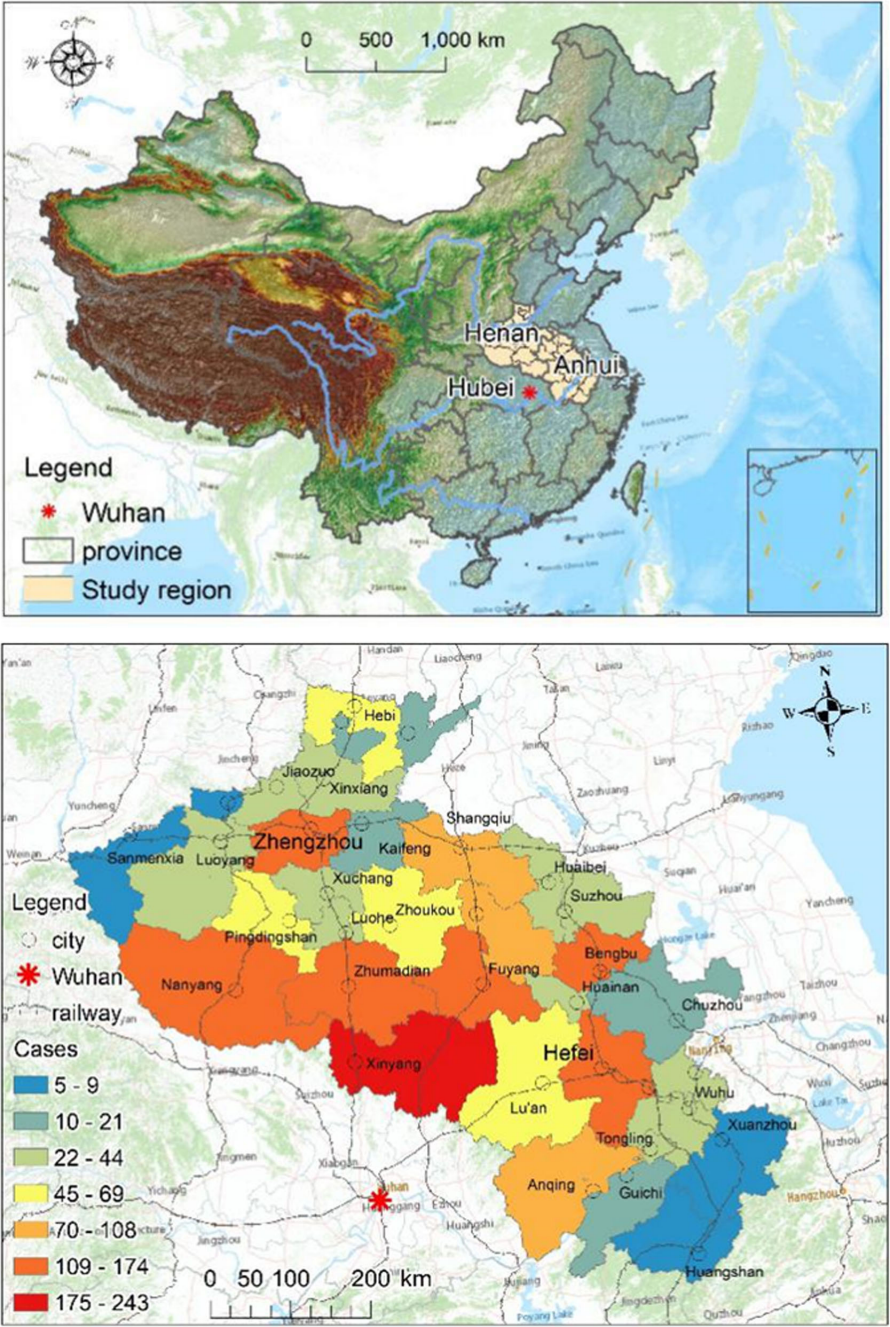
Geographic location of the Henan and Anhui provinces and the spatial distribution of COVID-19 cases

In this study, confirmed COVID-19 cases were collected from the websites of the Henan and Anhui provincial and municipal health commissions. A total of 2152 COVID-19 cases were collected in 34 municipal units from January 21 to February 24, 2020, including 1163 cases in Henan and 989 cases in Anhui (Fig. 1).

The availability of information and the representativeness of a time period are necessary conditions for research selection. Information about new coronavirus cases in Henan were first published on January 21, 2020. Since then, Henan and Anhui have begun to publish detailed case information. Of note, the number of dead during the study period was zero for three consecutive days before February 24, 2020.

**Fig. 2:**
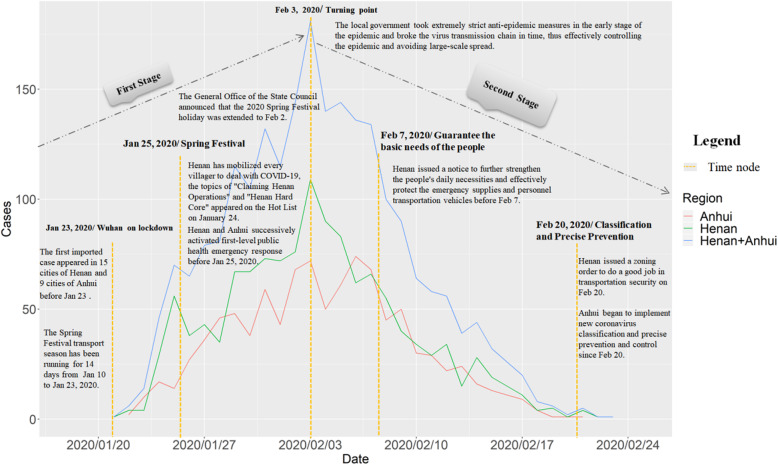
Time series distribution of COVID-19 daily new cases in Henan and Anhui

There were two distinct stages to the transmission of COVID-19 in the large labor export provinces: an outbreak period in the first stage and a control period in the second stage. These two stages were characterized by obvious increasing and decreasing trends during the epidemic period (Fig. 2).

The first stage can be divided into early and late periods. The early period of the first stage covered the Spring Festival transport season to when the Wuhan epicenter was placed on lockdown, between January 10 and January 23. The late period of the first stage spanned the Lunar New Year’s Eve to the Spring Festival holiday between January 24 and February 2.

The second stage covered February 2 to February 24. In this period, Henan and Anhui had passed the climax of the novel coronavirus outbreak. Daily new cases continued to run at high levels for more than ten days, but the epidemic had entered a controllable stage, and an overall decline was obvious after this peak.

In the study, the socioeconomic risks and protective factors included the urbanization rate, per capita GDP, proportion of primary industry GDP, doctors per 10,000 people, and proportion of the population that was more than 65 years old. Information regarding these factors was collected from the Henan and Anhui Statistical Yearbook. The population flow from Wuhan was a migration index, obtained from the Baidu map migration big data platform during the period between January 10 and January 23, 2020. The number of trains from Wuhan was calculated from the China Railway website (www.12306.cn). Table 1 shows the COVID-19 cases and various risk factors.

**Table 1:** Descriptive characteristics for various factors of COVID-19 cases

Variables	Minimum	Maximum	Mean	Median	Standard deviation
COVID-19 cases (person)	5	243	63	39	58
Population flow from Wuhan (%)	0.08	1.32	0.31	0.17	0.28
GDP (10,000 yuan)	600	9194	2127	1657	1710
Per Capita GDP (yuan)	1.94	9.31	4.76	4.33	1.93
Doctor per 10^3^ people	50.38	134.16	75.70	74.70	15.13
Population (10^4^ person)	73	1005	465	448	246
Population density (person /km^2^)	138	1156	582	644	251
Proportion of people older than 65 years old (%)	0.01	0.17	0.05	0.03	0.04
Proportion of primary industry GDP (%)	0.02	0.20	0.11	0.10	0.05
Urbanization (%)	39.77	73.75	52.81	51.94	8.92
Access to transportation (number of trains accessing the city)	0	126	20	4	31

**Fig. 3:**
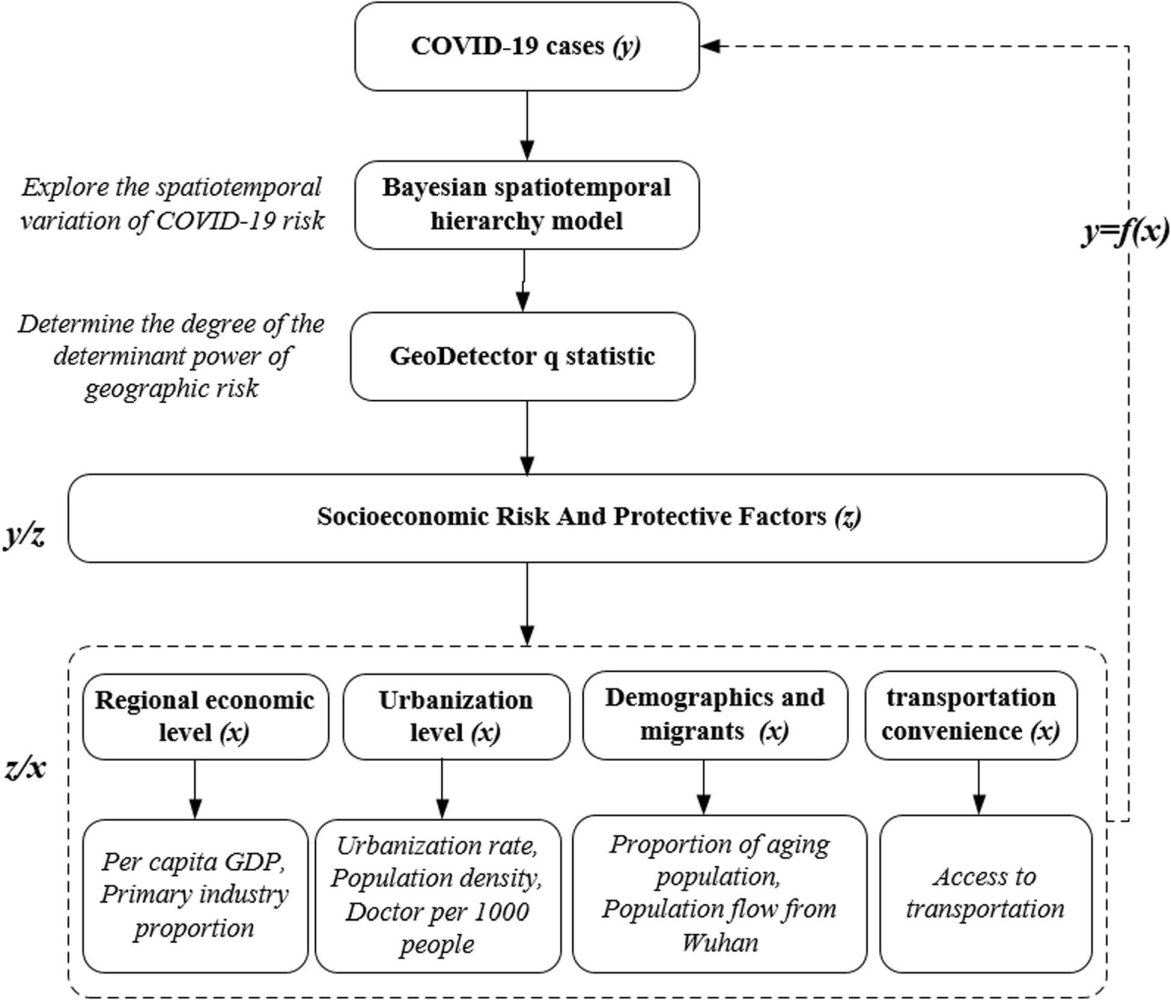
The relationship between the COVID-19 cases and their proxy variables. Note: *z* represents direct variables, and *x* characterizes indirect variables

The COVID-19 cases and all potential socioeconomic variables were calculated at the municipal level. Figure 3 shows the relationship between the COVID-19 cases and their proxy variables.

## Methods

### Bayesian spatiotemporal hierarchy model

A Bayesian spatiotemporal hierarchy model (BSTHM) in the current study was used to integrate population properties, sample information, and prior knowledge to assess the spatial and temporal relative risks and local trends in COVID-19.

The COVID-19 cases *y*_*it*_ in a city *i* and a day *t* were assumed to follow a Poisson distribution.

$$ {y}_{it}\sim Poisson\left({n}_{it},{r}_{it}\right), $$

where the parameters *n*_*it*_ and *r*_*it*_ represent the susceptible population and the relative risk of COVID-19 in city *i* in day *t*. The Poisson and log link regression functions were applied to assess the spatiotemporal variation of COVID-19 cases *y*_*it*_. The logarithmic transformation was expressed as the formula:

$$ \log \left({r}_{it}\right)=\alpha +{s}_i+\left({b}_0{t}^{\ast }+{v}_{\mathrm{t}}\right)+{b}_{1i}{t}^{\ast }+{\varepsilon}_{\mathrm{it}}, $$

where *α* is a constant term, the spatial term *s*_*i*_ represents the spatial relative risk of COVID-19 in city *i* relative to that risk in the whole region, the temporal term *b*_*0*_t* + *v*_*t*_ describes the overall temporal relative risk of the disease relative to that risk in the whole time length, t* indicates the centering time in the middle of the observation period, and the Gaussian noise *v*_*t*_ represents a random time effect. The term *b*_*1i*_ is the spatiotemporal interaction effect used to measure the deviation from the common spatiotemporal variation, describing the spatial heterogeneity of the temporal trend. The term *ɛ*_*1i*_ describes the variation not be interpreted in the model, which was assumed to be a Gaussian distribution in the study.

To further quantify the risk factors of COVID-19 that vary over space, time, or both based on the posterior estimated parameters of BSTHM, we used a two-stage method to identify the spatial relative risk (RR) and local temporal trends of COVID-19.

In the first stage, there were three classes for the spatial relative risk of COVID-19, stratified into hot spot, cold spot, or not hot/cold spot based on the posterior probability *p(exp (si) > 1 | data)*. A city was defined as a hot spot if the probability was greater than 0.8. A city was defined as a cold spot if the probability was less than 0.2. If not, a city was defined as neither a hot nor cold spot [18].

In the second stage, compared with the overall trend (*b*_*0*_t* + *v*_*t*_), the local temporal trend of each spot-class region *b*_*1i*_ was further stratified into faster, slower, or stable classes based on the posterior probability *p(b*_*1i*_ *> 0*|*h*_*i*_*, data)* in each city. A city was defined as a faster decreasing region if the probability was greater than 0.8, a city was defined as a slower region if the probability was less than 0.2, and a city was defined as a stable region if the probability was between 0.2 and 0.8 [18].

All of these processes were implemented into the WinBUGS software, and the posterior parameters were estimated through Markov chain Monte Carlo (MCMC) simulations [19].

### GeoDetector q statistic

In order to assess the spatial association between the potential socioeconomic factors and the overall spatial relative risk of COVID-19 calculated by BSTHM, the GeoDetector *q* statistic was used to determine the degree of the determinant power of the selected risk factors in the spatial stratified heterogeneity of the disease [20–22]. It was measured using the following formula:

$$ {\displaystyle \begin{array}{l}q=1-\frac{\sum \limits_{h=1}^L{N}_h{\sigma}_h^2}{N{\sigma}^2}\\ {}{\sigma}^2=\frac{1}{N}\sum \limits_{i=1}^N{\left({R}_i-\overline{R}\right)}^2\\ {}{\sigma_h}^2=\frac{1}{N}\sum \limits_{j=1}^{N_h}{\left({R}_{h,j}-{\overline{R}}_h\right)}^2\end{array}} $$

where *σ*^*2*^ and *σ*_*h*_^*2*^ represent the variance of the spatial relative risk of the disease in the whole region in *N* cities and in the *h*-th stratum in *N*_*h*_ cities, respectively. The parameters *R*_*i*_ and *R*_*h,j*_ represent the spatial relative risk in the *i*-th city and the *j*-th city in the *h*-th stratum, respectively. $\overline{R}$ and ${\overline{R}}_h$ refer to the average relative risk of the disease within the whole study region and a specific stratum, respectively. The *q* value ranged from 0 to 1. Higher values of the *q* statistic indicated a higher determinant power of the variable [20].

## Results

### Spatial relative risk of COVID-19

**Fig. 4:**
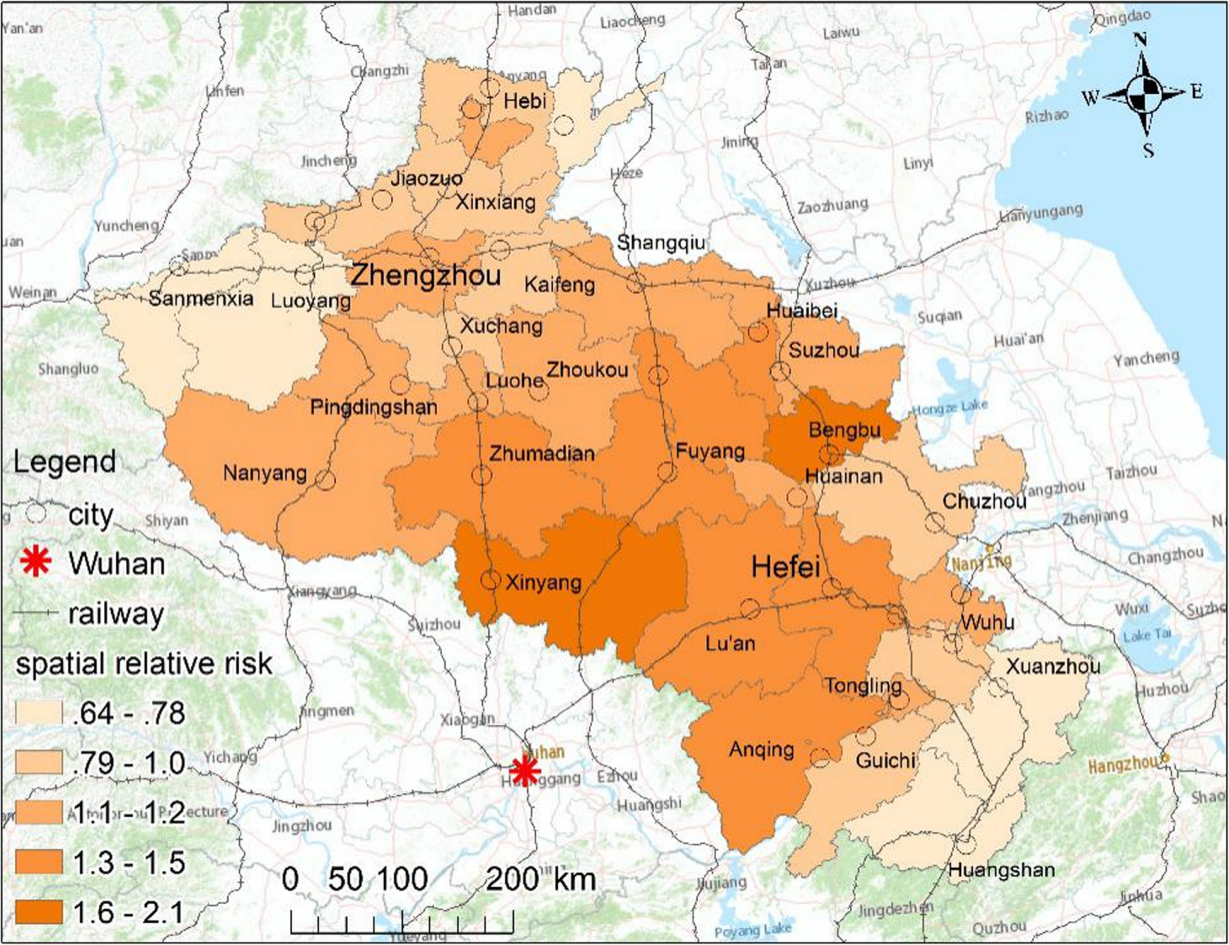
The posterior means of the overall spatial relative risks (exp(*s*_*i*_) of BSTHM)

Figure 4 shows the spatial relative risk *exp* (*s*_*i*_) of COVID-19 infection in the study region from January 21 to February 24, 2020. All of the cities presented different levels of risk according to their risk value *exp* (*s*_*i*_). This finding suggests that the infection risk of COVID-19 had a stable spatial heterogeneity.

The cities with the higher risk values of COVID-19 infection were located in two types of regions. One type included the southwest areas adjacent to Hubei, where the regional economy is relatively declining, there are few employment opportunities, the cities have large populations and are the main sources of outflows, and the cities have frequent and close social, economic, and transportation exchanges with Wuhan, as is the case in Xinyang. The other type included regions with big cities of concentrated populations that serve as economic and traffic hubs, such as Zhengzhou, Hefei, and Bengbu. Although they are far away from Hubei, the disease risk is significant due to the huge flow of returnees during the Spring Festival.

The overall spatial relative risk of COVID-19 had obvious and distinct temporal trends during the two stages of the study period. The spatiotemporal heterogeneity of the disease was analyzed separately for the first and second stages.

#### Spatiotemporal heterogeneity in the first stage

**Fig. 5:**
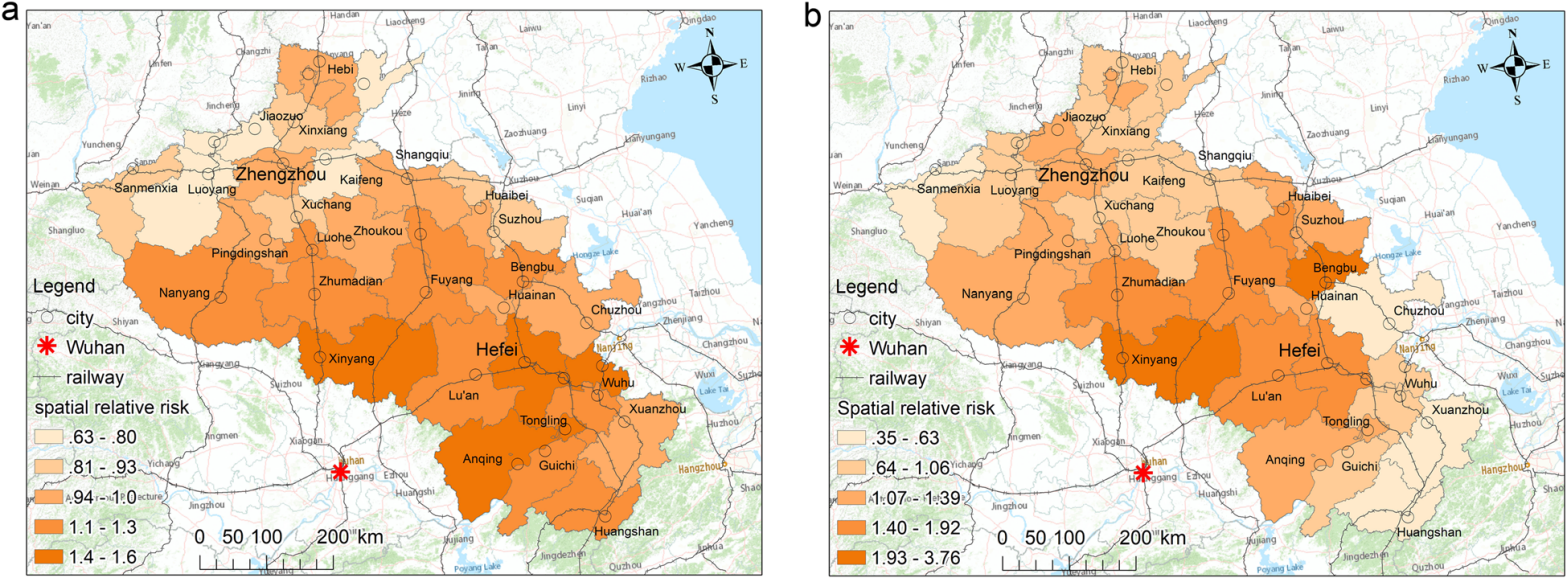
The spatial relative risks (exp(*s*_*i*_) of BSTHM) of COVID-19 in the (**a**) first and (**b**) second stage

Figure 5 shows the spatial relative risks in all of the cities during the first stage. The regions of low COVID-19 risk were mainly concentrated in the remote cities adjacent to Hubei province. This finding implied that the spatial risk of the disease in the first stage was mainly characterized by relocation diffusion.

**Fig. 6:**
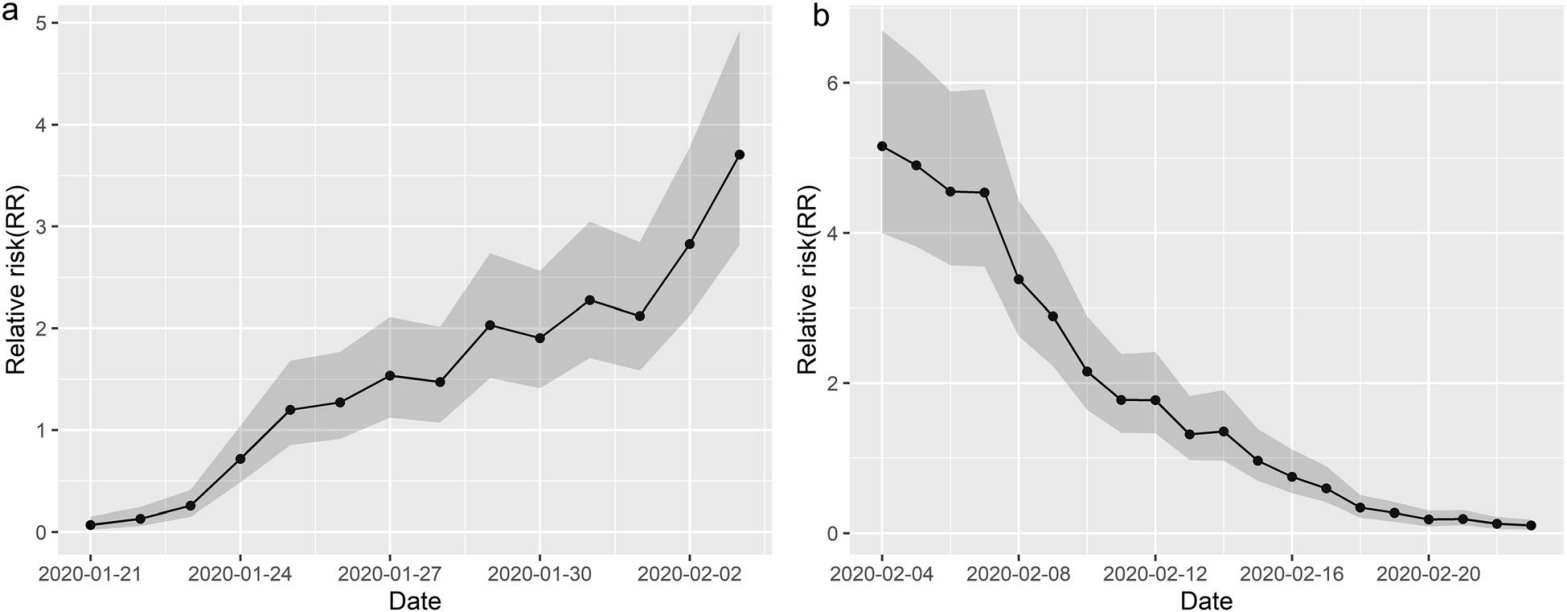
The COVID-19 RR temporal trend in the (**a**) first and (**b**) second stage (the posterior mean exp.(*b*_*0*_t* + v_*t*_) of BSTHM with a 95% CI)

The temporal relative risk of COVID-19 had an obvious upward trend in the first stage (Fig. 6). The results indicate that the relative risk of COVID-19 for all cities in the study region followed an increasing trend in the first stage. In January 21, 2020, the temporal RR was 0.06 with a confidence interval (CI) of [0.02, 0.15] at a significance level of 0.05. In February 3, 2020, the value was 3.71 with a CI of [2.82, 4.92] at a significance level of 0.05. Although the overall increasing trend in the first stage in the region was rapid, the local rising trends of COVID-19 risk varied in different cities.

**Fig. 7:**
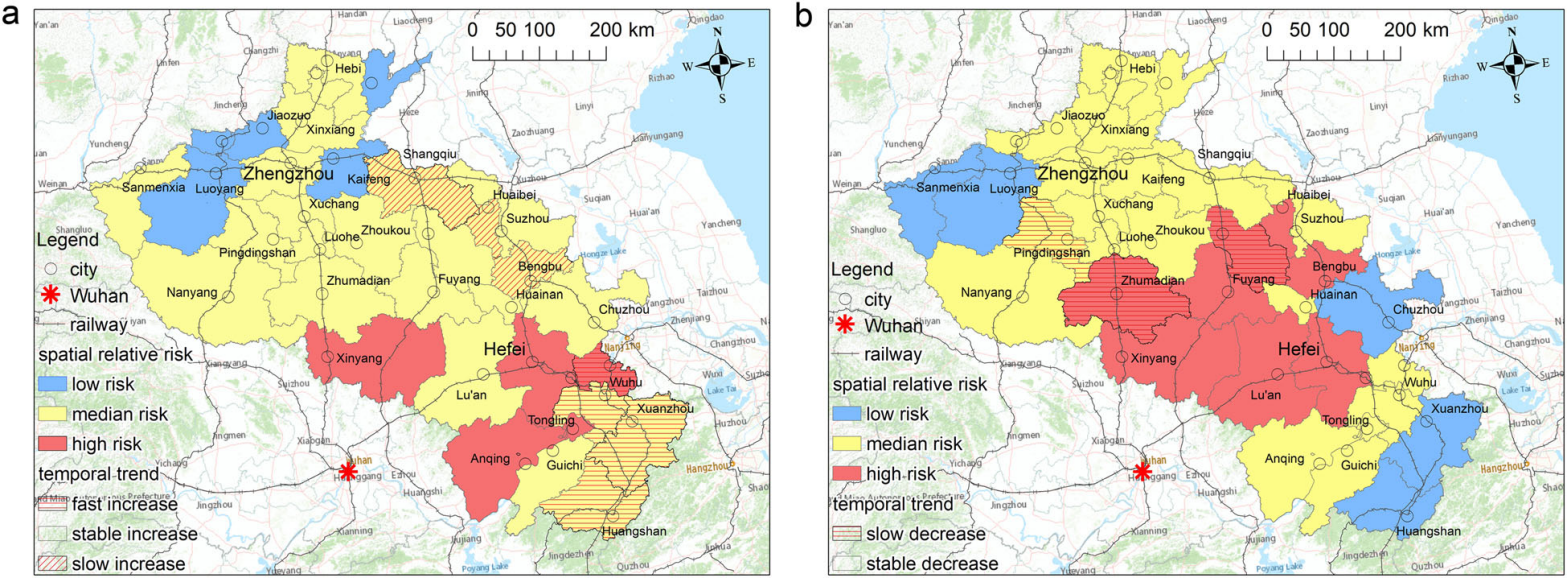
Hot/cold spot distribution of COVID-19 in the (**a**) first and (**b**) second stage

Figure 7 shows that all of the hot spot cities presented a faster and more stable temporal increase trend in comparison to the overall temporal trend. Thus, they may continue to maintain hot spot status over time. Meanwhile, 25% of the median-risk cities presented a slower or less stable increasing temporal trend in comparison to the overall temporal trend. Thus, they may become lower-risk cities or even convert into cold spots over time.

Table 2 presents the distribution of hot and cold spots of COVID-19 risk in the first stage. The results indicated that 14.71% of the cities were classified as both hot spots and cold spots in the stage, while the other 70.59% of counties were identified as neither a hot spot nor a cold spot. The cities in hot spots with a high spatial RR value were mainly located in the southwest area of the Henan province and central zone of the Anhui province.

**Table 2:** Cross-classification of COVID-19 risk in the first (I)and second (II) stage

Classification	Faster increase trend		Slower increase trend		Not different from common trend		Total	
Stage	I	II	I	II	I	II	I	II
Hot spots	1	0	0	2	4	6	5 (14.71%)	8 (23.53%)
Cold spots	0	0	0	0	5	5	5 (14.71%)	5 (14.71%)
Not hot/cold spots	3	0	3	2	16	19	24 (70.59%)	24 (61.76%)

#### Spatiotemporal heterogeneity in the second stage

Figure 5 shows the spatial relative risks in all of the cities during the second stage. The regions of low COVID-19 risk were mainly concentrated in the northeast Henan and southeast Anhui regions, which were far away from cities in the Hubei provinces. The regions of high COVID-19 infection risk encompassed all of the neighboring cities around Xinyang and several cities around Bengbu. This finding indicated that contagious diffusion occurred in the study region in the second stage.

The temporal relative risk of COVID-19 had a distinct downward trend in the second stage. The results showed that the relative risk for all cities in the study region followed a decreasing trend in the second stage (Fig. 6). In February 4, 2020, the COVID-19 temporal RR was 5.16 with a CI of [3.40, 6.70] at a significance level of 0.05. In February 24, 2020, the temporal RR was 0.10 with a CI of [0.05, 0.18] at a significance level of 0.05. Although the overall decreasing trend in the second stage in the region was rapid, the local downward trends of COVID-19 risk varied in different cities.

Figure 7 presents the local temporal trends regarding hot spots of COVID-19 risk in the second stage. Approximately 33% of the hot spot cities presented a slower temporal decrease trend in comparison to the overall temporal trend. Thus, they may remain hot spots and be high-risk. Meanwhile, 80% of the hot spot cities presented a temporal trend that was consistent with the common overall trend. These regions will also be high-risk in the future.

Table 2 presents the distribution of hot and cold spots of COVID-19 risk in the second stage. The results indicated that 23.53 and 14.71% of cities were classified as hot spots and cold spots during this stage, respectively. The other 61.76% of counties were identified as neither hot spots nor cold spots. Compared with the first stage, the cities in hot spots with high spatial RR values had spread farther into big cities, such as Hefei, the capital of Anhui.

### Risk factor detection

The *q* statistic results indicate that local economic and medical conditions play an important role in COVID-19 transmission (Table 3). The dominant socioeconomic factors were found to be related to the level of local economic development and industry. The risk of the disease was positively associated with a high proportion of primary industry, and the *q* statistic of the factor was 0.47. The disease risk was negatively associated with per capita GDP, and the *q* statistic of the factor was also 0.47. The other factors in the medical condition of a region, population density and urbanization, were also important influences on the spatial heterogeneity of the disease risk, and the *q* statistics of these factors were 0.45, 0.43, and 0.40, respectively (Fig. s1-s9).

**Table 3:** The determinant power of socioeconomic factors and their interactive effects on COVID-19 risk

Factors	Population flow from Wuhan	Per capita GDP	Doctor per 1000 people	Population size	Proportion of aging Population	Primary industry proportion	Urbanization	Access to transportation	Population density
Population flow from Wuhan	0.33								
Per capita GDP	0.70	0.47							
Doctor per 1000 people	0.73	0.69	0.45						
Population size	0.56	0.67	0.69	0.23					
Proportion of aging population	0.54	0.64	0.54	0.31	0.08				
Primary industry proportion	0.56	0.68	0.72	0.62	0.59	0.47			
Urbanization	0.64	0.63	0.65	0.57	0.56	0.63	0.40		
Access to transportation	0.51	0.74	0.72	0.68	0.63	0.55	0.6	0.32	
Population density	0.84	0.81	0.8	0.78	0.65	0.77	0.86	0.72	0.43

Likewise, the study found that population flow to Wuhan in Hubei province was an important influence on disease transmission. The *q* statistic of the population flow from Wuhan and the number of trains from Wuhan were 0.33 and 0.32, respectively.

In addition, the interaction effects on COVID-19 risk indicated that the coupled impact of population density and urbanization played an important role in COVID-19 spatial variation. The *q* statistic of this interaction relationship was 0.86. The interactive effects between population density and other risk factors were also great influences on COVID-19 risk. For example, the *q* statistic was 0.84 for the interaction between population density and population flow from Wuhan, 0.81 for the interaction between population density and per capita GDP, and 0.80 for the interaction between population density and the number of doctors per 1000 people (Table 3).

## Discussion

As of this time, the spatiotemporal variations and risk determinants of COVID-19 in typical labor export regions in China remain unclear. We used BSTHM as a novel two-stage method to explore the spatiotemporal variations of COVID-19 and applied the GeoDetector *q* statistic to quantify the determinant power of the risk factors and reveal the sources of heterogeneity underlying the patterns. The results showed that COVID-19 had significant spatiotemporal heterogeneity and that local economic and medical conditions, as well as population access to Wuhan in the Hubei province, played important roles in the transmission of COVID-19.

The early period of the first stage before the Lunar New Year on January 24, 2020 was characterized as an outbreak period, in which a huge number of people left Wuhan and returned home in the study region to reunite with their families for the New Year. The late period of the first stage between the Lunar New Year on January 24 and February 2, 2020, during the Spring Festival holiday, was characterized as the control period. In this period, families gathered for family reunion dinners on the Lunar New Year’s Eve. The duration and intensity of contacts occurring in the households increased the contagious transmission, which is now recognized as one important factor in the cluster transmission of the novel coronavirus [23].

In the second stage, with the epidemic developing, the spread of COVID-19 gradually shifted from an imported case pattern to a local case pattern. The greatest transmission risk existed in locations with low-detection capacity, high transportation, or economic connectivity to the epicenter of the outbreak [8]. This risk was usually related to the regions having large outflows into the surrounding areas of Hubei province, such as Henan and Anhui.

In this study, the risk of COVID-19 showed significant spatial heterogeneity in the study region. Relatively high-risk regions were mainly found in the areas adjacent to Hubei, while relatively low-risk regions were mainly located in the northeast Henan and southeast Anhui regions, far away from Hubei province. Therefore, the following three key points for the prevention and control of the COVID-19 epidemic in typical labor export provinces of China should be addressed. First, the critical areas were closely connected to socioeconomic transportation into and out of the epidemic area and neighboring areas. Second, transit cities and neighboring areas away from the epidemic center with big populations and heavy traffic. Third, some cities and neighboring areas with a large population of migrant workers returning from the epidemic area.

Previous studies have indicated that the transmission of an infectious disease is influenced by the characteristics of the virus and the susceptibility of the population, as well as their social and health conditions [6, 24–26]. Besides of these common factors, COVID-19 was also affected by some peculiar factors [8, 27, 28].

We found that some socioeconomic factors had significant associations with COVID-19 transmission. Some papers also have indicated that the persistence of infectious diseases is influenced by social and economic inequality [25]. These implied that imbalanced levels of regional economic development influenced the spread of COVID-19 in typical labor export provinces in China. These results were consistent with the findings from previous studies on other infectious diseases [29, 30].

The proportions of primary industry and per capita GDP were found to be strongly associated with the spatiotemporal variations of COVID-19 in the typical labor export provinces. These findings were consistent with other studies in the field of infectious diseases. For example, Li et al. found that rural areas show an increased risk of TB transmission due to poor economies and poor medical care [31]. Furthermore, Xu et al. found that the proportion of primary industry was positively associated with HBV incidences [32]. Agricultural areas contain many seasonal or long-term surplus populations who will seek work in the big cities and become migrant workers. Therefore, underdeveloped rural areas, as a source of migrant workers, need to become a focus for preventing and controlling imported cases.

In the present study, we also found that the number of doctors was strongly related to the COVID-19 risk in typical labor export provinces. Similar to our work, studies by Wang and Du showed that the abundance of medical resources and level of medical accessibility in a region play a vital role in the prevention of infectious diseases [33, 34]. Therefore, improving the abundance of medical resources and level of medical accessibility in a region is an effective measure to prevent and control the local spread of infectious diseases.

Population density, urbanization, and access to transportation were also strongly associated with COVID-19 transmission. These findings are consistent with previous studies [35–37]. A high population density is associated with a high disease risk. Communal activities in densely populated areas also increase the possibility of infectious disease outbreaks. Of note, urbanization had a nonlinear relationship with the disease outbreak. Rural areas of low urbanization were the source of migrant workers to Wuhan and had a high transmission risk. However, in areas of substantial urbanization, the greater concentration and connectedness of people and greater access to transportation also increased the risk of exposure to infectious disease and the speed of the spread of new infections.

In addition, the risk of COVID-19 infection was also significantly influenced by the population flow from Wuhan and the local age structure. These findings were consistent with those of previous studies [5, 6, 28, 38]. Therefore, effective measures to prevent or reduce disease transmission in typical labor export provinces should be implemented and studied in at-risk focus groups.

There were some limitations to this study. Firstly, the unique features of a local environment always give special characteristics to its inhabitants that will reflect on the spatiotemporal variations of COVID-19 risk. To our knowledge, COVID-19 risk presents spatial heterogeneity under socioeconomic conditions within a county or town area. Secondly, we focused on the COVID-19 risk in two provinces, which were mainly affected by the cases from their neighboring Hubei province, and in the study the train transportation facility was taken into account to determine the accessibility transportation, because train is the most important transportation for inter-provincial migration in China. Meanwhile, the short-distance migration was also affected by personal vehicle and bus transportation. In future studies, COVID-19 cases and related socioeconomic data at a finer spatial scale as well as the other transportation data will be collected to detect the relationship between COVID-19 and its risk factors.

## Conclusions

The findings of the present study show that COVID-19 had significant spatial heterogeneity in typical labor export provinces. The high-risk regions were mainly located in the big cities with concentrated populations that served as traffic hubs in areas adjacent to the outbreak epicenter in Hubei province. Local economic and medical conditions, as well as population access to Wuhan in Hubei province, played important roles in the transmission of COVID-19. These findings will be helpful for risk assessments of disease transmission and for implementing effective interventions to reduce the disease burden in provinces impacted by imported cases.

## Supplementary Information

**Additional file 1.** The relationship between COVID-19 risk and the socioeconomic risk factors.

## Data Availability

The datasets are available from the corresponding author upon reasonable request.

## Abbreviations

COVID-19: Coronavirus Disease 2019
GDP: Gross Domestic Product
SEIR: Susceptible, Exposed, Infectious, Removed
EIR: Exposed-identified-Recovered
BSTHM: Bayesian spatiotemporal hierarchy model
RR: relative risk
MCMC: Markov chain Monte Carlo
CI: confidence interval
