# Supplementary material for: Spatiotemporal heterogeneity and its determinants of COVID-19 transmission in typical labor export provinces of China
Source: BMC Infect Dis. 2021 Mar 5;21:242. doi: 10.1186/s12879-021-05926-x (PMC7935008; doi:10.1186/s12879-021-05926-x)
Supplement: Supplementary file 1 — Additional file 1. The relationship between COVID-19 risk and the socioeconomic risk factors. [file 12879_2021_5926_MOESM1_ESM.docx]

Additional file: **The relationship between COVID-19 risk and the socioeconomic risk factors**


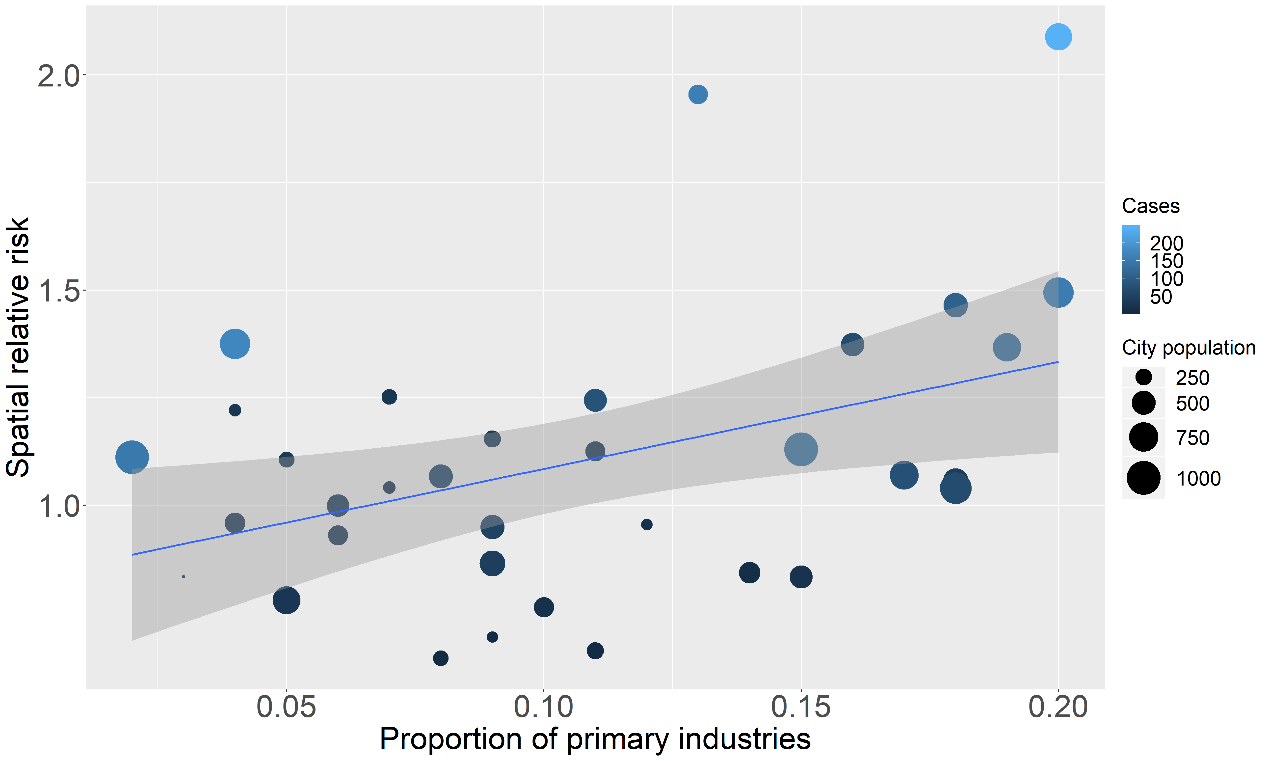


**Fig S1.** The relationship between COVID-19 risk and proportion of primary industry GDP


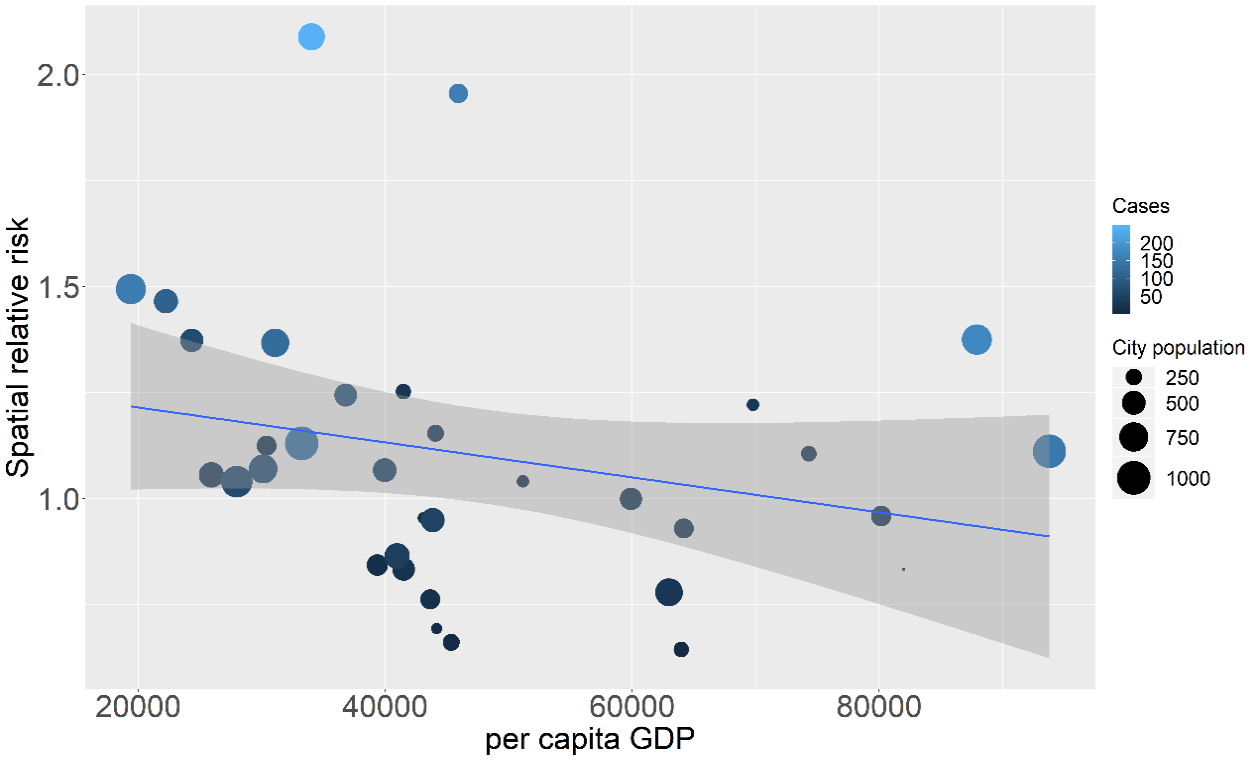


**Fig S2.** The relationship between COVID-19 risk and per capita GDP


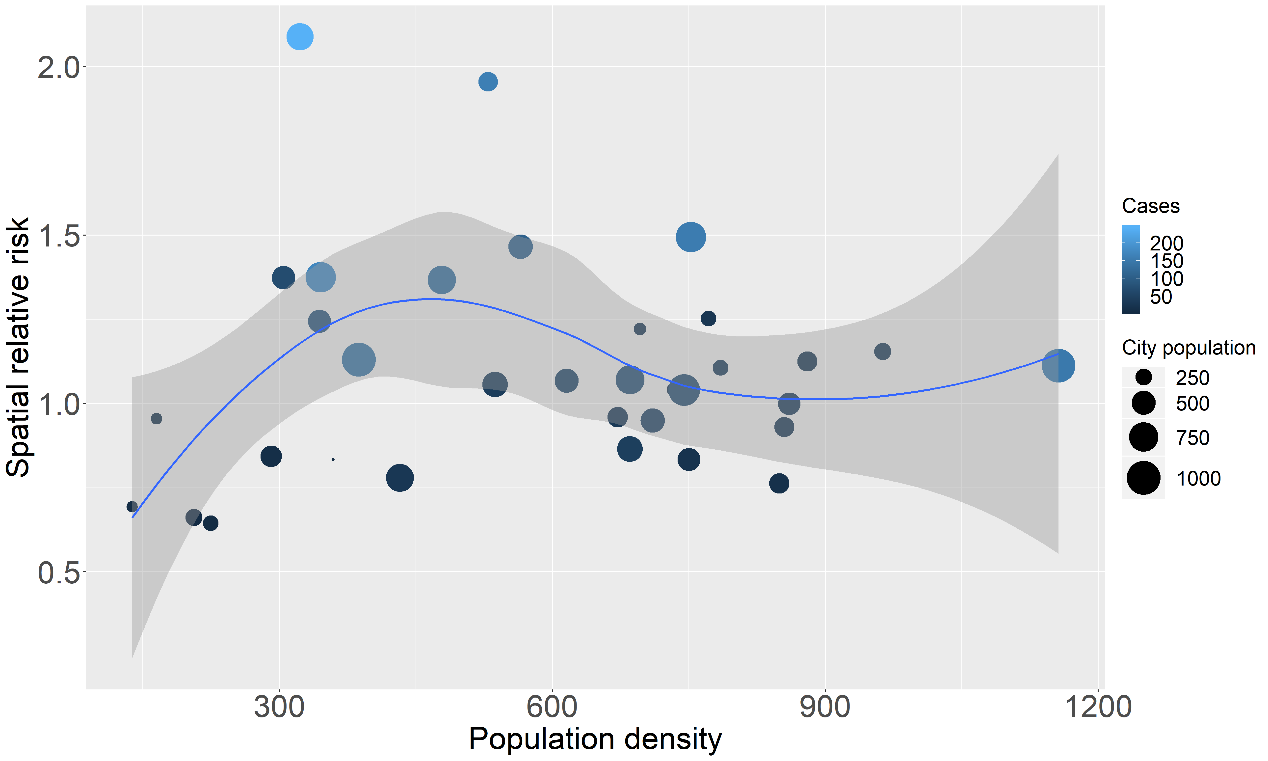


**Fig S3.** The relationship between COVID-19 risk and population density


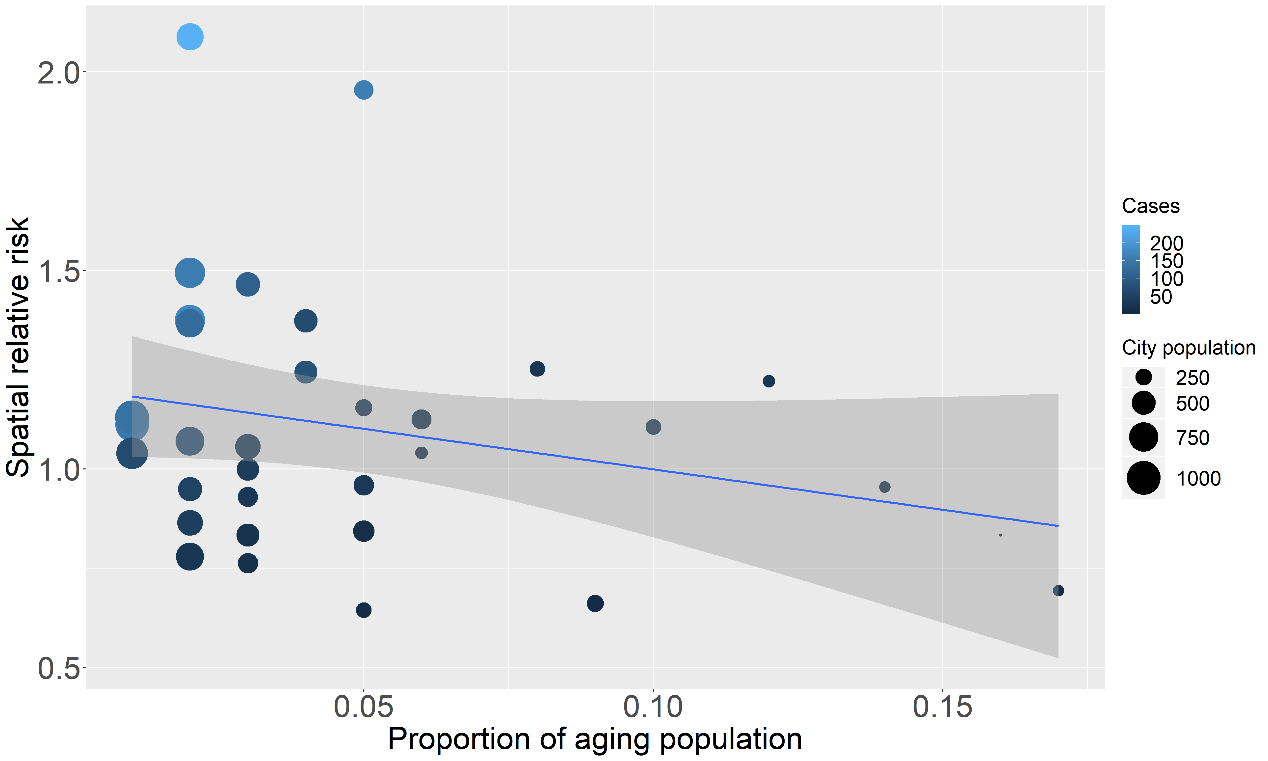


**Fig S4.** The relationship between COVID-19 risk and proportion of aging population


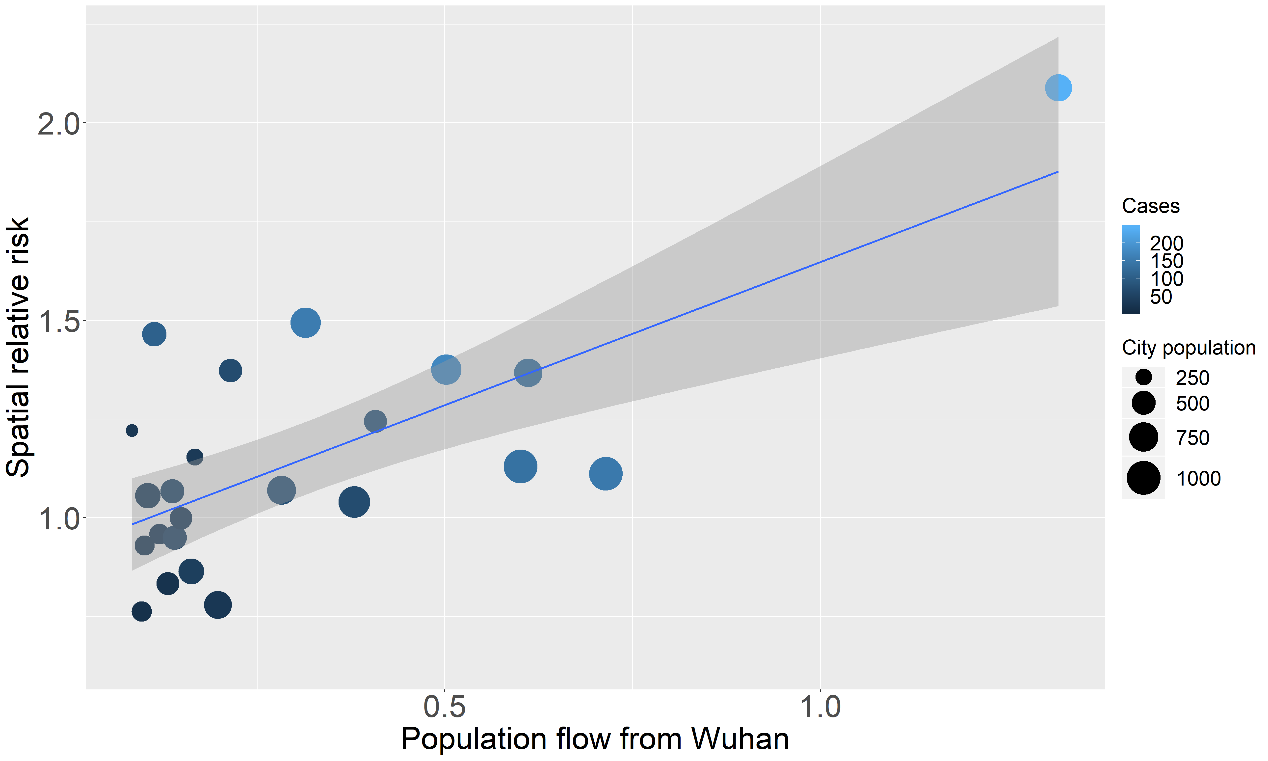
 **Fig S5.** The relationship between COVID-19 risk and population flow from Wuhan


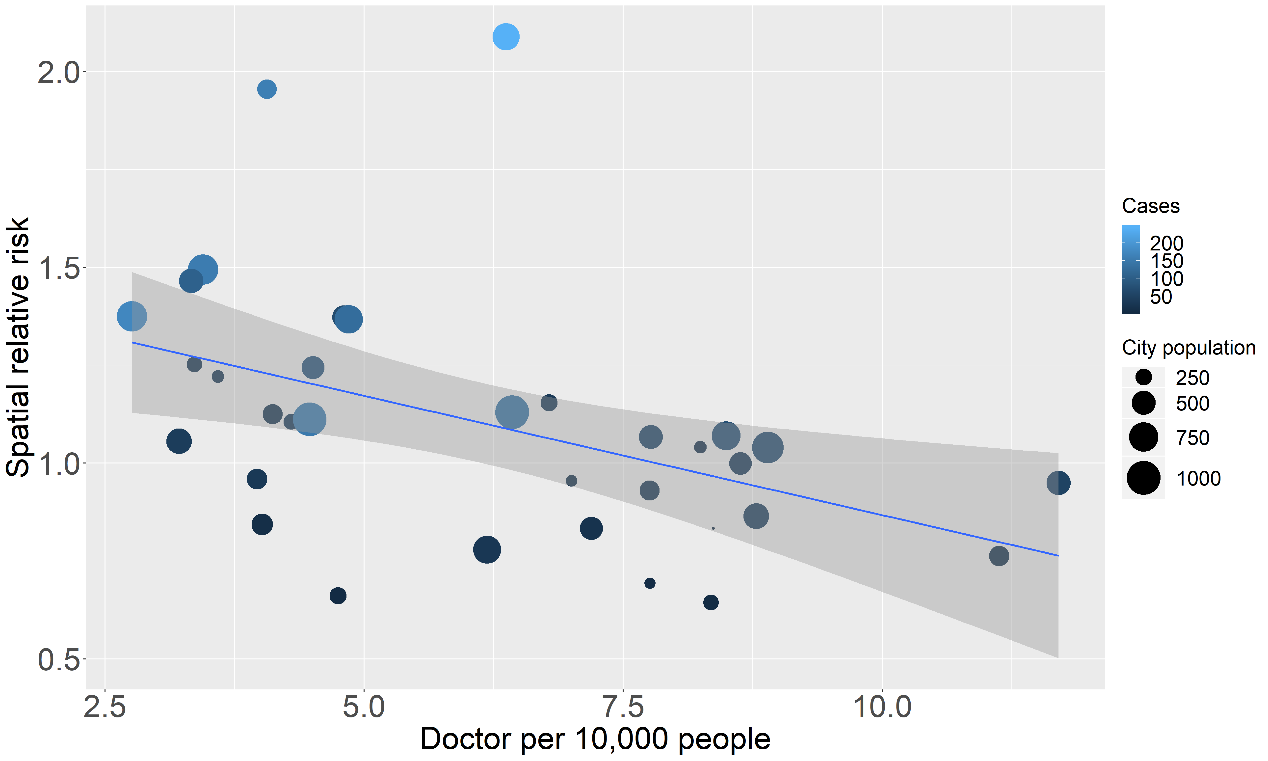


**Fig S6.** The relationship between COVID-19 risk and doctor per 1000 people


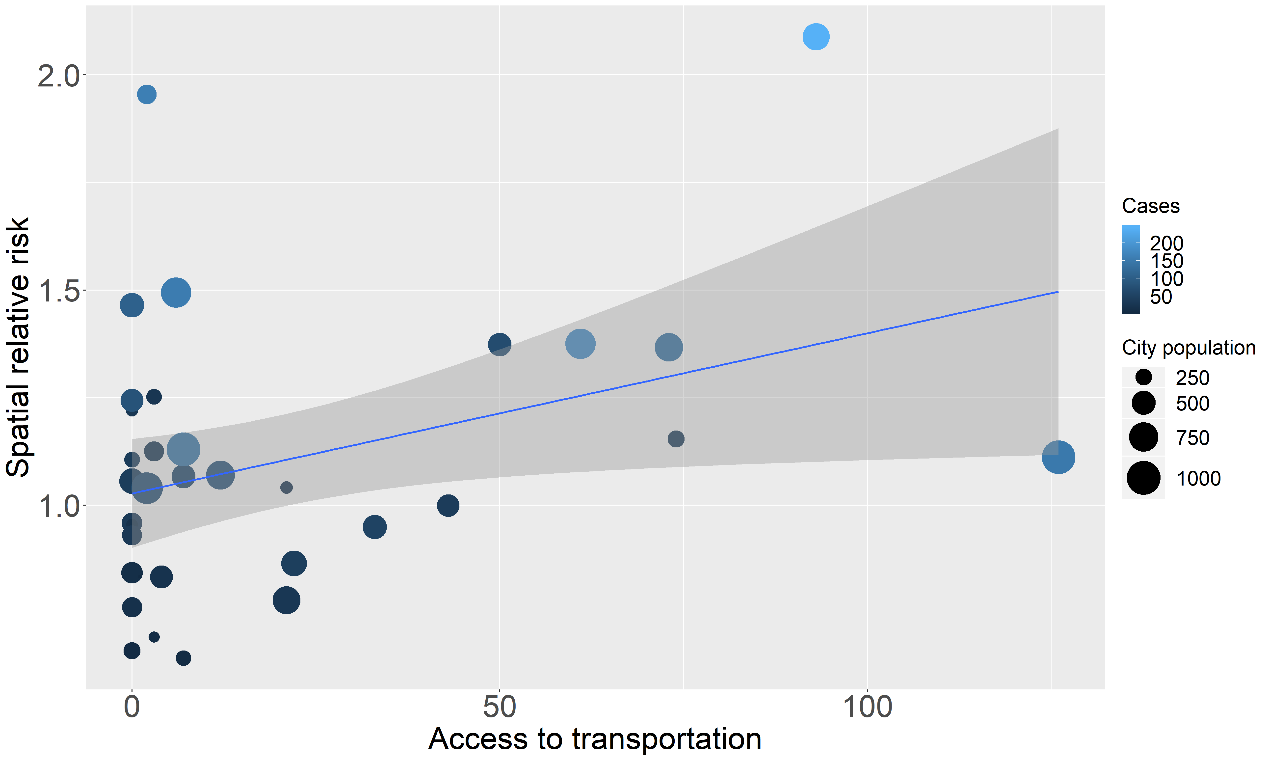
 **Fig S7.** The relationship between COVID-19 risk and access to transportation


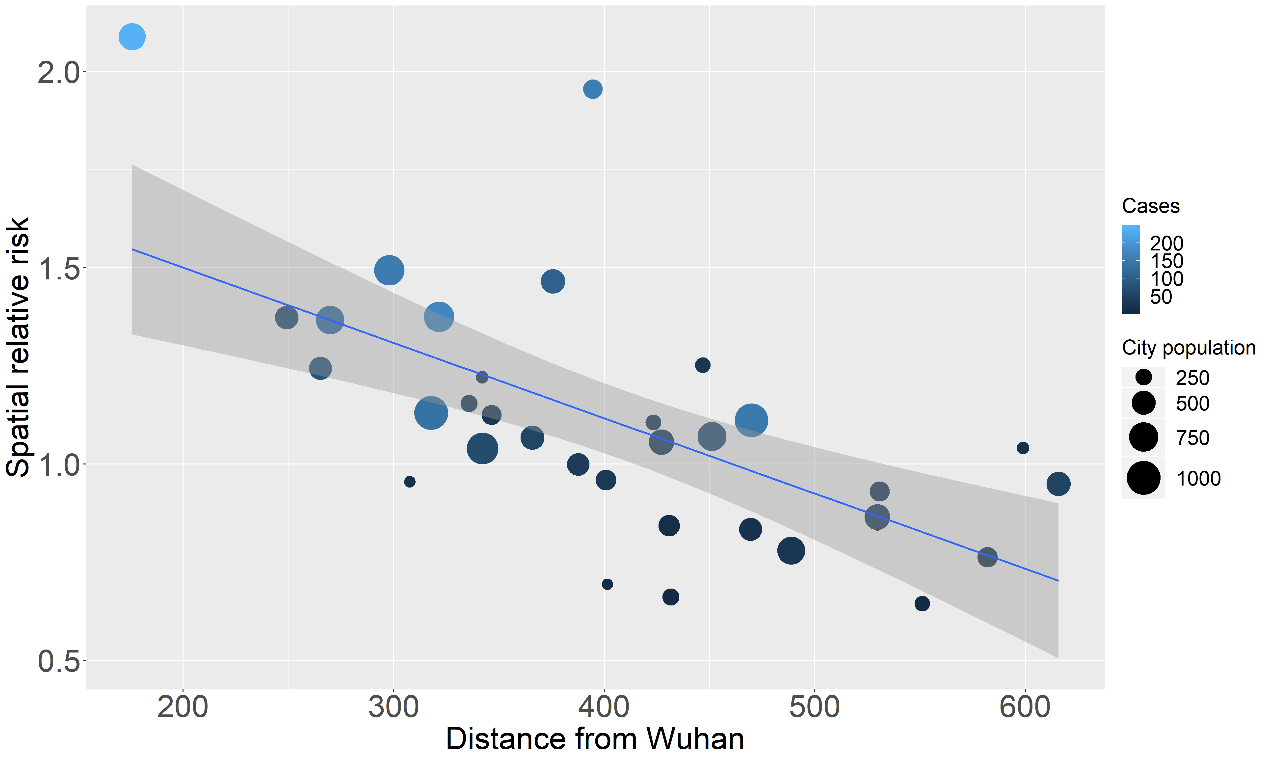


**Fig S8.** The relationship between COVID-19 risk and distance from Wuhan


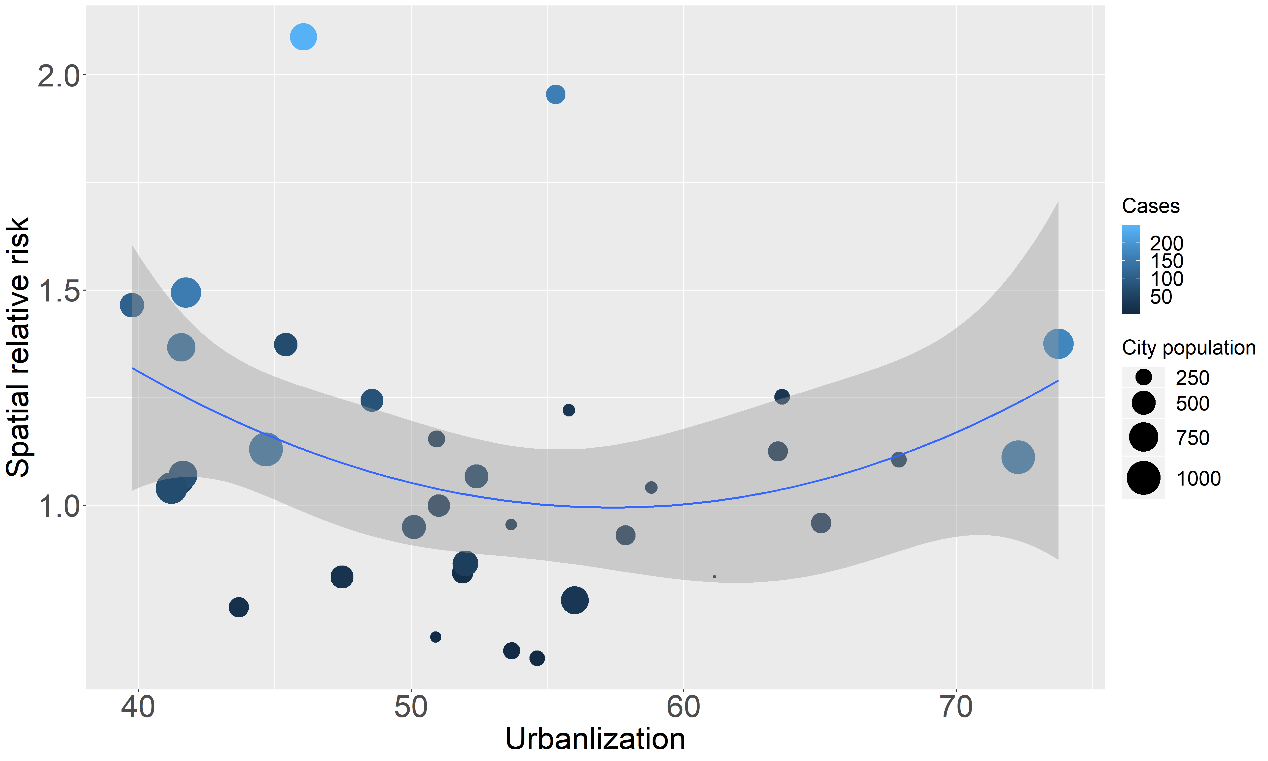


**Fig S9.** The relationship between COVID-19 risk and urbanization
